# The effect of *Qigong Wuqinxi* for osteopenia and primary osteoporosis: A protocol for systematic review and meta analysis

**DOI:** 10.1097/MD.0000000000020379

**Published:** 2020-05-22

**Authors:** Min Liu, Dongying Liu, Peipei Hong, Xianliang Qiu, Qiu Chen

**Affiliations:** Hospital of Chengdu University of Traditional Chinese Medicine, Chengdu, Sichuan Province, China.

**Keywords:** bone mineral density, osteoporosis, protocol, systematic review, Wuqinxi

## Abstract

**Introduction:**

Osteoporosis (OP) and related fragility fractures are a significant public health problem which leads to pain, disability, loss function of independence, considerable complications and increased mortality. Exercise training is the only alternative strategy to improve multiple skeletal and fall risk factors simultaneously. Wuqinxi is 1 of the Chinese mind-body exercises using to improve physical and mental health and fight against diseases for thousands of years. Our study aims to systematically review the existing literature to further explore the efficacy and safety of Wuqinxi in the prevention and treatment of osteopenia and OP.

**Methods and analysis:**

The following electronic databases (PubMed, Science Citation Index, Embase (Ovid) database, the Cochrane Library, the China National Knowledge Infrastructure, the China Biology Medicine disc, the China Science and Technology Journal Database, the Wan fang Database, ClinicalTrials.gov and the Chinese Clinical Trial Registry Platform) will be searched from the beginning to 1 June 2020. Only randomized controlled trials will be enrolled, in which the intervention group must include a form of Wuqinxi, while the control group can involve other conventional treatment or no intervention. The potential outcome measures will include bone mineral density values, bone turnover markers, fragility fractures, quality of life, pain scores, and adverse events. The Cochrane risk of bias assessment tool will be used to assess the risk of bias in each study.

**Results:**

The current study is a protocol for systematic review and meta-analysis without results, and data analysis will be carried out after the protocol. We will share our findings in the third quarter of 2021.

**Conclusion:**

This review aims to evaluate up-to-date evidence of Wuqinxi for bone health in English or Chinese language studies, and explore whether Wuqinxi can be used as an adjuvant treatment for osteoporosis and osteopenia.

**Ethics and dissemination:**

Ethical approval is not required as the review is a secondary study based on published literature. The results of the study will be published in peer-reviewed publications and disseminated electronically or in print.

**Protocol registration number:**

INPLASY202040135.

## 1 Introduction

### 1.1 Description of the condition

Osteoporosis (OP) is a growing global public health problem. It is associated with an increase in fragility fractures leading to pain, disability, loss function of independence, considerable complications and increased mortality.^[1]^ It is estimated that in the United States, among adults aged 50 years and older about 10.3% or 10.2 million had OP at the lumbar spine or femoral neck and 43.9% or 43.4 million had osteopenia at either skeletal site in 2010. When combined, the estimated number of adults with OP and osteopenia was 53.6 million, accounting for approximately 54% of the adult population aged 50 years and older in the US.^[2]^ OP-related fractures are a major and detrimental complication of OP. In the US over 1.7 million people were admitted to hospital with fragility fractures in 2011 and the direct costs associated with OP treatment exceeded $70 billion,^[3]^ imposing a heavy economic burden on individuals, families and healthcare systems.

### 1.2 What treatment modalities are important

In 2001, the National Institutes of Health defined OP as a skeletal disease characterized by decreased bone strength and increased fracture risk, suggesting that decreased bone mass is the main risk factor for osteoporotic fractures, but there are also other risk factors.^[4]^ Pharmaceutical agents targeting bone mineral density (BMD) are the first-line treatment for OP, yet patients often have poor compliance due to high medical costs, long-term treatments, and potential negative side effects.^[5,6]^ And Pharmaceuticals are not beneficial for other key fracture risk factors either, such as muscle strength and power, balance and agility, coordination and overall physical performance, all of which have been associated with a consequent increase in fall risks and susceptibility to fractures.^[7,8]^ Targeted exercise training is the only alternative strategy to improve multiple skeletal and fall risk factors simultaneously.^[9]^ Reviews of international evidence have shown that long-term progressive exercise can maintain and increase bone mineral content and musculoskeletal strength, improve balance and postural stability, decrease the risk of falls, and prevent the occurrence of fragility fractures.^[10–15]^

### 1.3 Description of the intervention

Wuqinxi was created by the well-known Chinese physician Huatuo based on the thoughts of “running water is never stale and a door-hinge never gets worm-eaten”, which means that regular exercises can help a person to keep good health. The movements of Wuqinxi originated from imitating the activity characteristics of five kinds of animals (tiger, deer, bear, ape, bird), and integrated with the combination of human body functions and the biological clock at the same time.^[16]^ Compared with conventional exercise modalities (such as strength training and resistance training), Wuqinxi is characterized by the harmonious combination of body movements, respiratory control and psychological adjustment together, so it can not only ease joints and stretch the human body but also help adjust the sub-health of the human body.^[17]^ A growing number of studies have shown positive effects of Wuqinxi on improving physical and psychological health,^[18]^ preventing and treating a variety of chronic diseases including hypertension, dyslipidemia,^[19]^ metabolic syndrome,^[20]^ knee osteoarthritis,^[21]^ and OP.^[22,23]^

### 1.4 The necessity of this review

Recent studies indicate that among the senile OP patients, Wuqinxi exercise is positive for bone metabolism and can effectively relieve and improve the symptoms of low back pain, it may also increase bone formation and decrease bone resorption to a certain extent.^[22,23]^ So far, the systematic review about the potential effect of Wuqinxi for primary OP was published in 2015,^[24]^ which only included 4 studies, suggested that Wuqinxi can improve OP-related pain symptom, but the effects on BMD and biochemical markers were uncertain owing to poor study design and execution, inconsistency, and imprecision. Further investigation is warranted given that an increasing number of studies about the effects of Wuqinxi on bone health have been carried out in recent years. Therefore, we will conduct an up-to-date systematic review and meta-analysis for existing randomized controlled trials (RCTs) with the aim of further assessing the effectiveness and safety of practicing Wuqinxi in the prevention and treatment of osteopenia and OP.

## 2 Materials and methods

The protocol of this systematic review will be reported following the Preferred Reporting Items for Systematic Review and Meta-Analysis Protocols checklist.^[25,26]^ This protocol has been registered with the International Platform of Registered Systematic Review and Meta-analysis Protocols (registration number: INPLASY202040135) which could be available on https://inplasy.com/.

### 2.1 Eligibility criteria

We will include studies according to the criteria outlined below

#### 2.1.1 Study designs

Only RCTs including combination therapy and monotherapy of Wuqinxi will be included. We will exclude letters to editors, review articles, case reports, conference abstracts, cross-sectional studies, and all observational studies.

#### 2.1.2 Participants

We will include studies on people who are osteopenia and primary OP or a population at high risk of OP (50 years or older). The clinical diagnosis of osteopenia and primary OP should be in accordance with internationally recognized criteria. For instance, the World Health Organization criteria: BMD of subjects evaluated by dual-energy X-ray absorptiometry could be categorized as: normal (T-score > -1); osteopenia, namely low bone mass (T-score in the range of -2.5 and -1); OP (T-score < or =-2.5).^[27]^

#### 2.1.3 Interventions

A comparison of Wuqinxi monotherapy against other treatments will be included, Wuqinxi plus another intervention versus the same intervention alone (eg, Wuqinxi and Calcium versus only Calcium) will be also enrolled. Any type of Wuqinxi will be included regardless of exercise version, frequency, and duration.

#### 2.1.4 Comparisons

The control group can receive a placebo, no treatment, vitamin D tablets, exercise or guideline-recommended conventional treatment. If the control group contains other non-conventional therapies, such as TaiChi, physiotherapy, herbal medicine, acupuncture, moxibustion, massage, yoga, it will be excluded.

#### 2.1.5 Outcomes

The potential outcomes of our interest contain the following:

Primary outcomes

(1) Changes in BMD values;
(2) Bone turnover markers, such as procollagen type 1 N-peptide and serum C-terminal telopeptide of type 1 collagen;
(3) OP-related fractures (fragility fractures).

Secondary outcomes

(1) Quality of life as measured by validated scales such as the short form -36;
(2) A recognized pain scores including the visual analog scale for pain (VAS pain);
(3) Any adverse events related to Wuqinxi for treatment or prevention during the trial.

### 2.2 Search methods

#### 2.2.1 Information sources

PubMed, Science Citation Index, Embase (Ovid) database, the Cochrane Library, and 4 Chinese databases (the China National Knowledge Infrastructure, the China Biology Medicine disc, the China Science and Technology Journal Database, and the Wan fang Database) will be searched from database inception to June 1, 2020. ClinicalTrials.gov and the Chinese Clinical Trial Registry Platform will be searched for ongoing or recently completed trials. Besides, we will scan the reference lists of included studies or relevant reviews to identify additional eligible studies, while the papers and unpublished reports will be hand-searched to ensure more complete coverage of the topic.

#### 2.2.2 Search strategies

A combination of medical subject headings and text words will be used to develop the literature search strategies, mainly including

(1) Wuqinxi, 5-Animal Exercise, five mimic-animal exercises, 5-animal boxing or Qigong;
(2) osteopenia, OP, postmenopausal osteoporosis, primary OP, senile OP, BMD, bone loss, low bone mass, bone∗turnover∗markers;
(3) clinical trial or randomized controlled trial.
  Two researchers (ML & DYL) will independently perform the literature search in the form of “back-to-back”, and only studies reported in English or Chinese language will be included due to resource limits.

Citations obtained from database searching will be managed using Endnote X7 software. We will present the initial draft of the search strategy with PubMed as an example (Table 1)

**Table 1:**
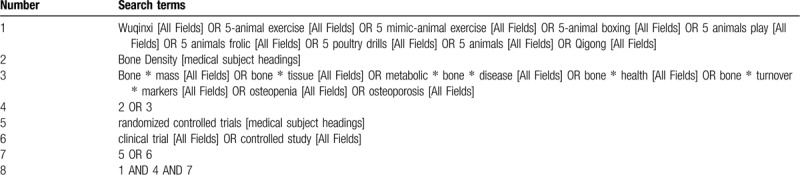
Example of PubMed search strategy. (Table 1 This table presents the initial draft of the search strategy with PubMed as an example.).

### 2.3 Data collection

#### 2.3.1 Selection of studies

**Figure 1:**
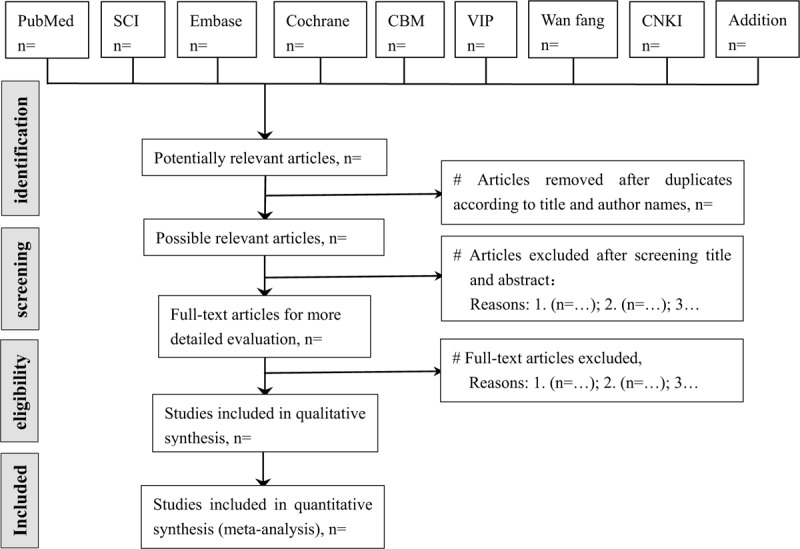
Study selection flow chart. SCI = science citation index; CBM = China biology medicine disc; VIP = China science and technology journal database; CNKI = China national knowledge infrastructure.

According to pre-defined eligibility criteria, the screening will be carried out in duplicate by 2 independent reviewers (ML & DYL) at each stage of the review. Studies will be removed if they don’t meet the inclusion criteria obviously. If the studies appear to meet the inclusion criteria or there is any uncertainty based on the information provided in the title and abstract, full texts will be obtained for further assessment. When necessary, we will contact the author for more details of the study to solve questions about eligibility. Disagreements will be resolved by discussion or consulting expert (QC) for arbitration. The number and reasons for excluding trials will be recorded in detail. A flow diagram of the study selection is shown in Figure 1.

#### 2.3.2 Data extraction

Data extraction for eligible studies will be performed independently by 2 reviewers (ML & DYL) using a pre-designed standardized form. We will provide guidance and interpretation for the contents of the extraction form before data extraction. The detailed data extraction form will mainly consist of basic information, population characteristics, methodological description, intervention characteristics, outcome data, conclusion and follow-up assessment. We will contact the original researchers for missing data. The third reviewer (PPH) will be responsible for checking the data extracted by the two reviewers. Inconsistencies will be resolved by discussion, and consulting the superior expert (QC) to facilitate the decision when a disagreement persisting.

### 2.4 Assessment of risk of bias

The methodological quality of individual studies will be judged following the criteria from the Cochrane Handbook for Systematic Reviews of Interventions Version 5.3.0.^[28]^ The judgments of all included studies will be made independently by two reviewers (ML & DYL), and we will conduct training of reviewers and calibration exercises before the start of the review to ensure consistency between reviewers. There are seven domains, each of which will be rated as “yes”(indicating a low risk of bias), “no”(indicating a high risk of bias), or “unclear”(indicating either an uncertainty for bias or lack of information). The original study investigators will be contacted if any uncertainty exists. We plan to compute graphic representations of potential bias within and across studies using Review Manager 5.3. Those with inconsistent opinions will be resolved through negotiation or consult the superior expert (QC) to reach a consensus. Overall, the following aspects will be considered:

(1) Appropriate generation of random allocation sequence (selection bias);
(2) Concealment of the allocation sequence (selection bias);
(3) Blinding of participants and healthcare providers (performance bias);
(4) Blinding of data collectors and outcome adjudicators (detection bias);
(5) Incomplete outcome data such as dropouts and withdrawals (attrition bias);
(6) Selective outcome reporting (publication or dissemination bias);
(7) Other bias (such as sponsorship bias).

### 2.5 Data analysis

#### 2.5.1 Data synthesis and meta-analysis

We will perform a systematic narrative synthesis to summarize and explain the characteristics and findings of the included studies and provide this information in the text and tables. Review Manager 5.3 provided by the Cochrane Collaboration will be used for the meta-analysis (If feasible), and the random-effects model will be chosen to combine all summary outcome measures. If a meta-analysis is impossible, the results of clinical trial comparisons will be analyzed descriptively. Dichotomous outcomes (eg, effective and ineffective) will be determined by relative risk with 95% confidence interval, while continuous data will be analyzed using weighted mean difference (if measurement methods are consistent) or standardized mean difference (if measurement methods are different).

#### 2.5.2 Dealing with missing data

When there are missing data, we will contact the study authors via email to obtain detailed accurate data. If the missing data are not available finally, we will carefully estimate the important numerical data, for example using an interpolation method. Moreover, the potential impact of missing data on the overall results of the study will be assessed using sensitivity analysis. It is possible to include multi-arm trials, we will combine the relevant groups into a single group according to the formula provided in the Cochrane handbook 5.3.0.^[28]^

#### 2.5.3 Assessment of heterogeneity and publication bias

Heterogeneity of each outcome measure will be tested using the *Chi*^*2*^ test and *I*^*2*^ statistic.^[29]^ If there is significant heterogeneity among the trials (*I*^*2*^≥50% or *P* < .1), we will try to explain the source of heterogeneity through subgroup analysis or sensitivity analysis. And we should not perform a meta-analysis if heterogeneity is substantial, a narrative qualitative summary will be done instead. Funnel plot will be used to reveal potential publication bias if over 10 studies are available.^[30]^

#### 2.5.4 Subgroup analysis and sensitivity analysis

Subgroup analysis will be further stratified by type of subjects (elderly, postmenopausal women), diagnosis (OP, low bone mass), BMD at different skeletal regions (lumbar spine, femoral or total hip), treatment type, or co-interventions. To explore the robustness of our meta-analysis, we will compare the results before and after by removing one study each time and then pooling the remaining studies. When the heterogeneity test suggests *I*^*2*^ < 50% or *P* > .1, we will compare whether the results are consistent after replacing the random-effects model with a fixed-effect model in the meta-analysis.

### 2.6 Grading the quality of evidence

The quality of evidence in the systematic review will be judged by the Grading of Recommendations Assessment, Development, and Evaluation tool.^[31]^ It is based on five key domains: risk of bias, consistency, directness, precision and publication bias. The evidence levels for each outcome will be adjudicated as high quality, moderate quality, low quality, and very low quality.^[32]^ RCTs with low risk of bias are considered high-quality evidence that could provide a direct and precise reference for clinical application.

### 2.7 Reporting of the review

The methodological quality of the systematic review and meta-analysis to be completed next will be standardized by each item of the A measurement tool to assess systematic reviews 2 tool.^[33]^ And the results will be reported following the Preferred Reporting Items for Systematic Reviews and Meta-Analysis statement published in 2009.^[34]^

## 3 Discussion

Wuqinxi, as one of the important components of traditional Chinese mind-body exercise, has been used to improve physical and mental health and fight against diseases for thousands of years. Its style is slow, gentle, easy and convenient to practice, not limited by time, place, different age levels or exercise equipment, and can be popularized and applied in communities. However, prior findings regarding the Wuqinxi for preventing and treating osteopenia and OP are inconsistent, as the latest and most comprehensively updated systematic review and meta-analysis, this study will fill the gap in the literature and further summarize the effects of Wuqinxi on bone health.

The strengths of our study are that firstly, a comprehensive search of existing Chinese and English databases and grey literature libraries will be performed to ensure that all relevant literature is captured as much as possible. Secondly, the study selection, data extraction and assessment of the risk of bias will be completed in duplicate by 2 independent reviewers at each stage of the review. Training of reviewers and calibration exercises will be conducted before the start of the review to ensure consistency between reviewers, and any disagreements will be resolved by discussion or consulting expert for arbitration. Thirdly, only randomized controlled trials will be included in this study, which improves the quality of evidence for outcome measures. Lastly, the study will be completed in strict accordance with the entries in the A measurement tool to assess systematic reviews 2 to improve the methodological quality.

Our study may have some potential limitations. First of all, Wuqinxi is a traditional practice exercise in which participants and healthcare providers can’t be blinded so that the performance bias may have been introduced. And second, RCTs to be included in our study may be performed in various populations and complex clinical settings therefore the risk of potential heterogeneity will be present. Finally, although we will employ a broad search strategy to minimize publication bias, some language bias may exist owing to restrictions of language. However, because of the importance of the topic, our study could be used as a reference for future related research.

As mentioned above, this systematic review and meta-analysis will conducive to determine the potential benefits and safety of Wuqinxi for the prevention and treatment of OP. Furthermore, the findings of the study may not only provide a reference for the revision of the guidelines, but also could promote the application of traditional Chinese exercise worldwide (such as Wuqinxi, Taichi, Baduanjin), which would benefit more people in the future.

## Author contributions

**Conceptualization:** Min Liu

**Data curation:** Xianliang Qiu, Peipei Hong

**Formal analysis:** Min Liu, Dongying Liu

**Methodology:** Min Liu

**Project administration:** Min Liu, Qiu Chen

**Software:** Dongying Liu, Peipei Hong

**Supervision:** Qiu Chen

**Validation:** Dongying Liu

**Writing – original draft:** Min Liu, Dongying Liu

**Writing – review & editing:** Min Liu, Qiu Chen

QC is the guarantor. All authors read, provided feedback and approved the final manuscript.

## References

[1] Osteoporosis prevention, diagnosis, and, therapy. JAMA 2001;285:785–95. 11176917 10.1001/jama.285.6.785

[2] Wright NC Looker AC Saag KG The recent prevalence of osteoporosis and low bone mass in the United States based on bone mineral density at the femoral neck or lumbar spine. J Bone Miner Res 2014;29:2520–6. 24771492 10.1002/jbmr.2269 PMC4757905

[3] Ogdie A Nowell WB Applegate E Patient perspectives on the pathway to psoriatic arthritis diagnosis: results from a web-based survey of patients in the United States. BMC Rheumatol 2020;4:2. 31938764 10.1186/s41927-019-0102-7 PMC6953285

[4] NIH consensus development panel on osteoporosis prevention, diagnosis, and therapy, march 7–29, 2000: highlights of the conference. South Med J 2001;94:569–73. 11440324

[5] Crandall CJ Newberry SJ Diamant A Comparative effectiveness of pharmacologic treatments to prevent fractures: an updated systematic review. Ann Intern Med 2014;161:711–23. 25199883 10.7326/M14-0317

[6] Kanis JA Svedbom A Harvey N The osteoporosis treatment gap. J Bone Miner Res 2014;29:1926–8. 24956507 10.1002/jbmr.2301

[7] Kothawala P Badamgarav E Ryu S Systematic review and meta-analysis of real-world adherence to drug therapy for osteoporosis. Mayo Clin Proc 2007;82:1493–501. 18053457 10.1016/S0025-6196(11)61093-8

[8] Daly RM Dalla VJ Duckham RL Exercise for the prevention of osteoporosis in postmenopausal women: an evidence-based guide to the optimal prescription. Braz J Phys Ther 2019;23:170–80. 30503353 10.1016/j.bjpt.2018.11.011 PMC6429007

[9] Cawthon PM Fullman RL Marshall L Physical performance and risk of hip fractures in older men. J Bone Miner Res 2008;23:1037–44. 18302496 10.1359/JBMR.080227 PMC2679379

[10] Lord SR Ward JA Williams P The effect of a 12-month exercise trial on balance, strength, and falls in older women: a randomized controlled trial. J Am Geriatr Soc 1995;43:1198–206. 7594152 10.1111/j.1532-5415.1995.tb07394.x

[11] Lord SR Lloyd DG Nirui M The effect of exercise on gait patterns in older women: a randomized controlled trial. J Gerontol A Biol Sci Med Sci 1996;51:M64–70. 8612105 10.1093/gerona/51a.2.m64

[12] Judge JO Whipple RH Wolfson LI Effects of resistive and balance exercises on isokinetic strength in older persons. J Am Geriatr Soc 1994;42:937–46. 8064101 10.1111/j.1532-5415.1994.tb06584.x

[13] Campbell AJ Robertson MC Gardner MM Randomised controlled trial of a general practice programme of home-based exercise to prevent falls in elderly women. BMJ 1997;315:1065–9. 9366737 10.1136/bmj.315.7115.1065 PMC2127698

[14] Liu-Ambrose TY Khan KM Eng JJ The beneficial effects of group-based exercises on fall risk profile and physical activity persist 1-year post-intervention in older women with low bone mass: follow-up after withdrawal of exercise. J Am Geriatr Soc 2005;53:1767–73. 16181178 10.1111/j.1532-5415.2005.53525.x PMC3377605

[15] Wayne PM Kiel DP Krebs DE The effects of Tai Chi on bone mineral density in postmenopausal women: a systematic review. Arch Phys Med Rehabil 2007;88:673–80. 17466739 10.1016/j.apmr.2007.02.012

[16] Li XF Research on the Development of Our Health Qigong Associations in Present Stage [master degree] [master, thesis]. Beijing: Beijing Sport University; 2008.

[17] Guo Y Xu M Wei Z Beneficial effects of Qigong Wuqinxi in the improvement of health condition, prevention, and treatment of chronic diseases: evidence from a systematic review. Evid Based Complement Alternat Med 2018;2018:3235950. 30473716 10.1155/2018/3235950 PMC6220394

[18] Chen X Wang DM Health Qigong Wuqinxi improves hydrogen proton magnetic resonance spectra in prefrontal cortex and hippocampus in college students with mild depression. J South Med Univ 2016;36:1468–76. 27881335

[19] Li ZW Zhou LJ Observation on patients with dyslipidemia treated by five-animal exercises. J Guangzhou Sport Univ 2009;29:97–103.

[20] Liu HM Correlation research on effect of Wuqinxi exercise on cognitive function in old people with metabolic syndrome. J Wuhan Inst Physic Edu 2012;46:56–61.

[21] Tu P Liao YP Effect of Wuqinxi and Zhanzhuang on knee Ffexor and extensor strength and WOMAC scores of female patients with KOA. J Chengdu Sport Univ 2014;40:68–84.

[22] Shen MR Feng YJ Wei T Effect of Hua Tuo's frolics of five animals on the bone mineral density of lumbar vertebrae in senile patients with osteoporosis. Chin J Osteoporosis 2013;19:271–4.

[23] Shen MR Feng YJ Wei WW Effect of HUA Tuo's Frolics of five animals on the patients with senile osteoporosis. Chin J Tradit Chin Med Pharm 2014;29:895–7.

[24] Wei X Xu A Yin Y The potential effect of Wuqinxi exercise for primary osteoporosis: a systematic review and meta-analysis. Maturitas 2015;82:346–54. 26386831 10.1016/j.maturitas.2015.08.013

[25] Moher D Shamseer L Clarke M Preferred reporting items for systematic review and meta-analysis protocols (PRISMA-P) 2015 statement. Syst Rev 2015;4:1. 25554246 10.1186/2046-4053-4-1 PMC4320440

[26] Shamseer L Moher D Clarke M Preferred reporting items for systematic review and meta-analysis protocols (PRISMA-P) 2015: elaboration and explanation. BMJ 2015;350:g7647. 25555855 10.1136/bmj.g7647

[27] Kanis JA Melton LJ Christiansen C The diagnosis of osteoporosis. J Bone Miner Res 1994;9:1137–41. 7976495 10.1002/jbmr.5650090802

[28] Higgins JPT, Green S, eds. Cochrane handbook for systematic reviews of interventions version 5.3.0: The Cochrane Collaboration, 2015. Available at: http://handbook-5-3.cochrane.org/ (updated Oct 2015).

[29] Higgins JP Thompson SG Deeks JJ Measuring inconsistency in meta-analyses. BMJ 2003;327:557–60. 12958120 10.1136/bmj.327.7414.557 PMC192859

[30] Hayashino Y Noguchi Y Fukui T Systematic evaluation and comparison of statistical tests for publication bias. J Epidemiol 2005;15:235–43. 16276033 10.2188/jea.15.235 PMC7904376

[31] Guyatt GH Oxman AD Vist GE GRADE: an emerging consensus on rating quality of evidence and strength of recommendations. BMJ 2008;336:924–6. 18436948 10.1136/bmj.39489.470347.AD PMC2335261

[32] Balshem H Helfand M Schünemann HJ GRADE guidelines: 3. Rating the quality of evidence. J Clin Epidemiol 2011;64:401–6. 21208779 10.1016/j.jclinepi.2010.07.015

[33] Shea BJ Reeves BC Wells G AMSTAR 2: a critical appraisal tool for systematic reviews that include randomised or non-randomised studies of healthcare interventions, or both. BMJ 2017;358:j4008. 28935701 10.1136/bmj.j4008 PMC5833365

[34] Moher D Liberati A Tetzlaff J Preferred reporting items for systematic reviews and meta-analyses: the PRISMA statement. PLoS Med 2009;6:e1000097. 19621072 10.1371/journal.pmed.1000097 PMC2707599

